# Heart rate cut-offs to identify non-febrile children with dehydration and acute kidney injury

**DOI:** 10.1007/s00431-022-04381-3

**Published:** 2022-01-29

**Authors:** Pierluigi Marzuillo, Anna Di Sessa, Dario Iafusco, Daniela Capalbo, Cesare Polito, Felice Nunziata, Emanuele Miraglia del Giudice, Paolo Montaldo, Stefano Guarino

**Affiliations:** 1 Department of Woman, Child and of General and Specialized Surgery, Università Degli Studi Della Campania “Luigi Vanvitelli, ” Via Luigi De Crecchio 2, Naples, Italy; 2 Department of Pediatrics, AORN Sant’Anna E San Sebastiano, via Ferdinando Palasciano, Caserta, 81100 Italy; 3 Centre for Perinatal Neuroscience, Imperial College London, London, UK

**Keywords:** Dehydration, Heart rate, Acute kidney injury, Children

## Abstract

We hypothesized that the heart rate (HR) variation in an acute setting compared with HR in wellbeing status could be a good marker of both dehydration and acute kidney injury (AKI). Since HR in wellbeing status is unknown in most cases, we assumed as reliable surrogate the 50th percentile of HR according to age and gender. We evaluated if the estimated percentage of heart rate variation in acute setting compared with 50th percentile of HR (EHRV) could be marker of dehydration and AKI in children. Two independent cohorts, one prospective comprehending 185 children at type 1 diabetes mellitus onset (derivation) and one retrospective comprehending 151 children with acute gastroenteritis and pneumonia (validation), were used to develop and externally validate EHRV as predictor of the ≥ 5% dehydration and/or AKI composite outcome. Febrile patients were excluded. EHRV was calculated as ((HR at admission–50th percentile of HR)/HR at admission) × 100. The prevalences of ≥ 5% dehydration and AKI were 61.1% and 43.8% in the derivation and 34.4% and 24.5% in the validation cohort. For the ≥ 5% dehydration and/or AKI composite outcome, the area under receiver-operating characteristic curve of the EHRV in the derivation cohort was 0.69 (95%CI, 0.62–0.77; *p* < 0.001) and the best EHRV cut-off was > 24.5%. In the validation cohort, EHRV > 24.5% showed specificity = 100% (95%CI, 96.2–100.0), positive predictive value = 100%, and negative predictive value = 67.1% (95%CI, 64.7–69.5). The positive likelihood ratio was infinity, and odds ratio was not calculable because all the patients with EHRV > 24.5% showed ≥ 5% dehydration and/or AKI.

*   Conclusions*: EHRV appears a rather reliable marker of dehydration and AKI. Further validations could allow implementing EHRV in the clinical practice.

**Table:** 

**What is Known:** *• Increased heart rate (HR) is an easily and quickly detectable sign of dehydration in childhood, but its cut-off to suspect dehydration or acute kidney injury (AKI) is not defined.*	
**What is New:** *• We found that a percentage of estimated HR variation in acute setting in comparison with 50th percentile of HR (EHRV)>24.5% predicted ≥5% dehydration and/or AKI in non-febrile patients.* • *We provide a one-page tool to suspect ≥5% dehydration and/or AKI on the basis of the HR. If furtherly validated, this tool could be implemented in the daily clinical practice.*

**Supplementary information:**

The online version contains supplementary material available at 10.1007/s00431-022-04381-3.

## Introduction

Gastroenteritis and dehydration account for a large proportion of childhood hospitalizations [1]. Among California children aged 1 through 5 years, gastroenteritis and dehydration accounted for 10.6% of hospital discharges, ranking second to asthma, which accounted for 12.8% [2].

Early recognition of dehydration signs and proper definition of dehydration degree could improve the care of these patients and could reduce hospital admissions for dehydration [3].

The gold standard to classify the dehydration is the percentage of weight loss [4]. Therefore, parents’ perceptions of their children’s weight before the event which determines dehydration would be important to properly define the dehydration degree and to choose the best rehydration modality. Parents, however, are often unable to discriminate the weight status of their children [5].

Clinical parameters that can support in the evaluation of a child with suspect of dehydration are capillary refill > 2 s, dry mucous membranes, absent tears, generally ill appearance, deep breathing, and increased thirst [6]. Unfortunately, with the exception of capillary refill time, these parameters are highly subjective, and it is not possible to define objectifiable cut-off values [6].

While clinicians may feel that they can intuitively identify which children are severely dehydrated and which ones are not, general practice physicians and nurses are likely to both over- and under-diagnose severe dehydration based on their overall clinical impression, suggesting an important role for the use of standardized scales when assessing children with suspect of dehydration [7].

Increased heart rate (HR) is the first mechanism of compensatory adaptation to hypovolemia [8], and it is a recognized sign of dehydration in childhood [9]. However, although it is an easily and quickly detectable sign, HR cut-off values to define dehydration at present are missing [9].

Acute kidney injury (AKI) is often a consequence of renal hypoperfusion due to dehydration in children [10–12]. We hypothesized that HR variation in an acute setting compared with HR in wellbeing status could be a good clinical marker of both dehydration and AKI.

Since HR in wellbeing status is an unknown parameter in most cases, we assumed as reliable surrogate the 50th percentile of HR according to age and gender [13]. We aimed to evaluate whether the estimated percentage of heart rate variation in acute setting compared with 50th percentile of HR according to age and gender (estimated heart rate variation, EHRV) could be a reliable marker of dehydration and AKI in children. The EHRV accuracy and performance have been first assessed in a derivation cohort of children with onset of type 1 diabetes mellitus (T1DM) and then validated in an independent dataset of children (external validation).

## Methods

### Derivation cohort

The HR-based index to identify children with dehydration or AKI was developed by a post hoc analysis of the DiAKIdney (T1DM and AKI) cohort [10], a prospectively enrolled cohort of 185 patients with T1DM onset in which weight, HR, and serum creatinine were evaluated both at T1DM onset and again after 14 days when they had fully recovered the acute phase [10]. This allowed us to precisely measure the percentage of weight loss and HR variation in acute setting (at T1DM onset) compared with wellbeing status (in absence of any acute event, after 14 days). Moreover, at admission for T1DM onset, we asked parents an estimate of their children’s weight loss. We chose this group of patients as derivation cohort because T1DM onset represents a good model of dehydration in children.

### External validation cohort

We validated the findings of the derivation cohort in a separate cohort of children affected by acute gastroenteritis [11] and community acquired pneumonia [14] enrolled in a different hospital.

This cohort comprehends 300 patients, 114 (38%) with acute gastroenteritis [11], and 186 (62%) with community acquired pneumonia [14]. After the exclusion of 149 febrile patients (26 among patients with gastroenteritis and 123 among those with community acquired pneumonia), a total of 151 children were enrolled. Out of the 151 enrolled patients, 88 (58.3%) presented with acute gastroenteritis and 63 (41.7%) with community acquired pneumonia.

Due to the retrospective nature of this cohort — unlike the derivation cohort — precise details about the dehydration degree were not available. Therefore, we validated our findings only based on the presence of ≥ 5% dehydration and AKI.

More in detail, the dehydration was classified in < 5%, or ≥ 5% of fluid deficit on the basis of retrospective evaluation of clinical conditions reported in clinical charts and according to the World Health Organization definition [4]. This definition evaluates the dehydration degree on the basis of some clinical parameters including general appearance (ranging from well to lethargic), eyes (normal or sunken), thirst (not thirsty, thirsty, drinks poorly, or not able to drink), and skin turgor (skin goes back quickly, slowly, or very slowly). A patient with no dehydration (< 5% of fluid deficit) should be in well general condition, with normal eyes, not thirsty, and with skin that goes back quickly. If the patient has 2 signs among irritability, sunken eyes, thirsty, and skin turgor that goes back slowly is at least moderately dehydrated (≥ 5% of fluid loss). In addition to this definition, we considered a patient as at least moderately dehydrated if underwent intravenous fluids administration.

For the AKI definition, we considered as basal serum creatinine the value of creatinine estimated using previously validated back-calculation methods [15]. The Hoste(age) equation was used to back-calculate basal serum creatinine [11, 14], assuming that basal eGFR were the median age-based eGFR normative values for the children ≤ 2 years of age [16], and eGFR = 120 mL/min/1.73 m^2^ for children > 2 years [17, 18].

### Definitions

The weight was always evaluated in kilograms.

Percentage of estimated weight loss (EWL) = [(estimated weight − weight at T1DM onset)/estimated weight]*100.

Estimated weight = estimated (from parents) weight loss at T1DM onset + weight at admission.

Percentage of measured heat rate variation (MHRV) in acute setting compared with wellbeing status = ((HR at admission − HR after 14 days)/HR at admission) × 100.

EHRV = [(HR at admission − 50th percentile of HR for age and sex)/HR at admission]*100. We used the percentile charts provided by Sarganas et al. [13] in children aged ≥ 3 years, otherwise those provided by Fleming et al. [19]

AKI was defined according to the Kidney Disease/Improving Global Outcomes (KDIGO) serum creatinine and/or urine output criteria [20]. We considered as basal the serum creatinine values obtained at the last follow-up visit when all the biochemical parameters showed normalization and estimated glomerular filtration rate (eGFR) were within normal range for age [21].

According to World Health Organization, the dehydration was classified as mild in case of < 5%, moderate in case of 5–10%, and severe in case of > 10% of MWL [4].

### Statistical analysis

Details about power calculation and statistical analyses are shown in the Supplementary Text. In brief, the post hoc power was > 90%. Linear regression, Spearman test, logistic regression, and receiver-operating characteristic (ROC) curve analyses were used to analyse the data presented in this manuscript. The Youden index was used to identify the best cut-offs at ROC curve analysis [22]. Moreover, we calculated sensitivity, specificity, accuracy, positive and negative likelihood ratio, positive and negative predictive value (PPV and NPV), and odds ratio (OR) of the cut-offs identified at ROC curve analyses.

We calculated the crude HR value for each age which should increase the clinical suspicion of ≥ 5% dehydration and/or AKI as follows: 50th percentile of HR for age and sex + ((50th percentile of HR for age and sex/100) × the best EHRV cut-off).

The study was approved by our Research Ethical Committee (protocol number 0014355/i/2020), and all parents provided written informed consent before any procedure.

## Results

### Derivation cohort

The mean age of derivation cohort was 9.1 years (4.1 SDS). None of the patients in this group was febrile or presented pain. Out of 185 patients, 72 (38.9%) presented with mild, 61 (32.9%) with moderate and 52 (28.2%) with severe dehydration. AKI was found in 81/185 patients (43.8%) [10]. Out of 72 patients with mild, 61 with moderate, and 52 with severe dehydration, 20 (27.8%), 28 (45.9%), and 33 (63.5%) patients (< 0.001) presented AKI, respectively. The mean EHRV was 22.8% (13.9 SDS).

**Fig. 1:**
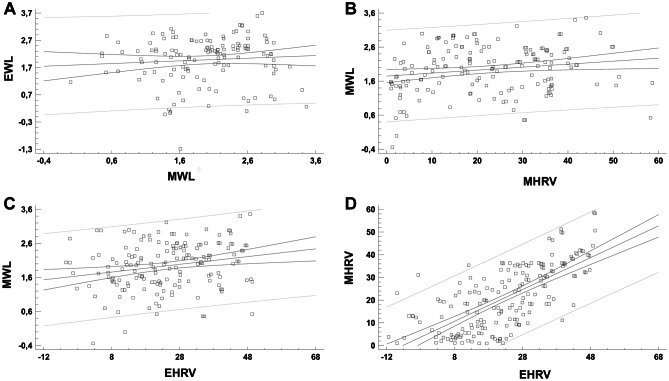
**A** Regression analysis describing the relationship between EWL and MWL. Model r2 = 0.6%; *p* = 0.37; correlation coefficient = 0.07. The regression is described by the equation *y* = 1.78757 + 0.0980459*x. *p* value for intercepts was < 0.001; *p* value for the slopes was 0.37. Spearman test: *r* = 0.12, *p* = 0.16. **B** Regression analysis describing the relationship between MWL and MHRV. Model r2 = 8.4%; *p* < 0.001; correlation coefficient = 0.29. The regression is described by the equation *y* = 1.37961 + 0.20342*x. *p* value for intercepts was < 0.001; *p* value for the slopes was < 0.001. Spearman test: *r* = 0.26, *p* < 0.001. **C** Regression analysis describing the relationship between MWL and EHRV. Model r2 = 5.9%; *p* = 0.002; correlation coefficient = 0.24. The regression is described by the equation *y* = 1.17894 + 0.250065*x. *p* value for intercepts was < 0.001; *p* value for the slopes was 0.002. Spearman test: *r* = 0.31, *p* < 0.001. **D** Regression analysis describing the relationship between MHRV and EHRV. Model r2 = 48.6%; *p* < 0.001; correlation coefficient = 0.70. The regression is described by the equation *y* = 5.08734 + 0.700665*x. *p* value for intercepts was < 0.001; *p* value for the slopes was < 0.001. Spearman test: *r* = 0.70, *p* < 0.001

Linear regression analysis showed no significant relation between EWL and MWL (*p* = 0.37) (Fig. 1A). On the other hand, we found a significant relation between MWL and MHRV (*p* < 0.001) (Fig. 1B), between MWL and EHRV (*p* = 0.002) (Fig. 1C), and between MHRV and EHRV (*p* < 0.001) (Fig. 1D).

**Fig. 2:**
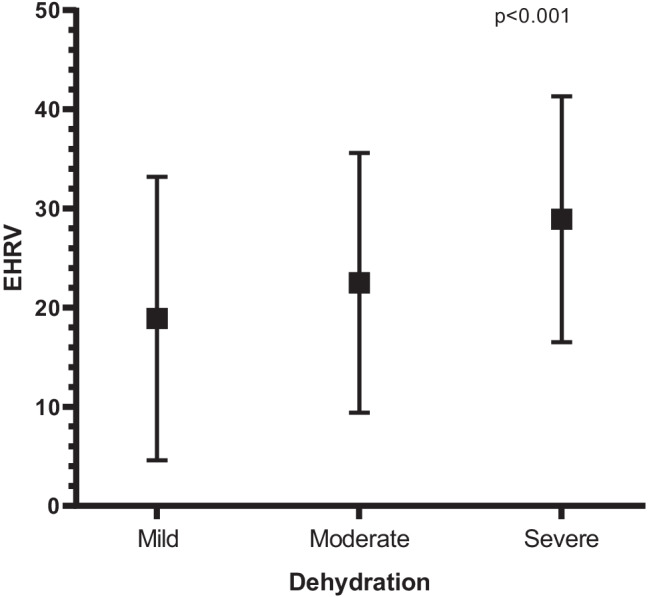
Means and SDS of EHRV among patients with mild, moderate, and severe dehydration. For mild, moderate, and severe dehydration, the means (SDS) were 18.9 (14.3), 22.5 (13.1), and 28.9 (12.4), respectively

Patients with mild had lower EHRV than patients with moderate dehydration. These latter, in turn, showed lower EHRV than patients with severe dehydration (overall *p* < 0.001) (Fig. 2).

**Fig. 3:**
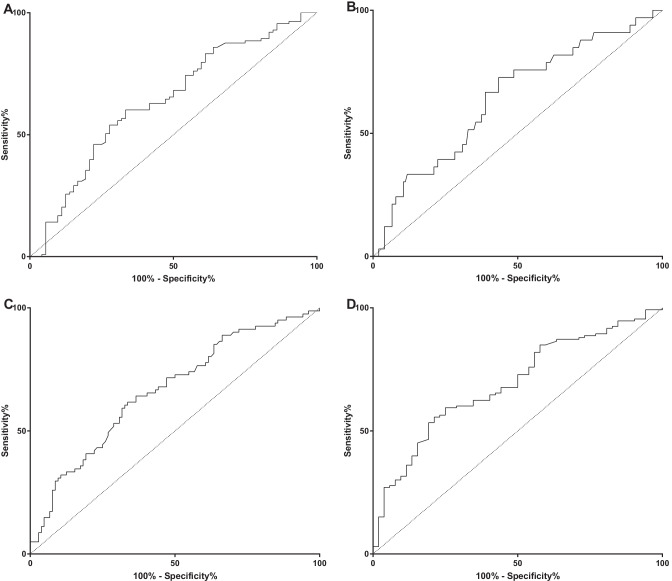
ROC curve analyses of the derivation cohort. **A** ROC curve for ≥ 5% dehydration. **B** ROC curve for > 10% dehydration. **C** ROC curve for AKI. **D** ROC curve for composite outcome defined by ≥ 5% dehydration and/or AKI

Both univariate and multivariate logistic regression analyses showed that EHRV (as continuous linear variable) was a significant prognostic factor for ≥ 5% dehydration, > 10% dehydration, and AKI (Supplementary Table 1). The EHRV had a significant AUROC for ≥ 5% dehydration (AUROC = 0.64; 95%CI: 0.56–0.71; *p* = 0.001) (Fig. 3A), > 10% dehydration (AUROC = 0.67; 95%CI: 0.60–0.74; *p* < 0.001) (Fig. 3B), and for presence of AKI (AUROC = 0.67; 95%CI: 0.59–0.73; *p* < 0.001) (Fig. 3C).

Based on the Youden test, the best cut-off values were EHRV > 23.2% for ≥ 5% dehydration, EHRV > 23.8% for > 10% dehydration, and EHRV > 24.5% for AKI. However, these cut-off values were very similar and hence difficult to use in the daily clinical practice. Therefore, we decided also to evaluate the accuracy of EHRV as predictor of the composite outcome of ≥ 5% dehydration and/or AKI. The ≥ 5% dehydration and/or AKI composite outcome was defined by the presence of either ≥ 5% dehydration or AKI or both. The AUROC was 0.69 (95%CI: 0.62–0.77; *p* < 0.001) (Fig. 3D). The best cut-off value for this composite outcome was EHRV > 24.5%. The prognostic accuracy of the cut-offs identified at the ROC curve analyses are shown in the Supplementary Table 2.

### External validation cohort

The mean age of external validation cohort was 2.9 years (2.5SDS). None of these patients presented with pain. Out of 151 patients, 52 (34.4%) showed ≥ 5% dehydration and 37 (24.5%) AKI. Patients with ≥ 5% dehydration and AKI respectively showed higher EHRV levels compared with patients without ≥ 5% dehydration (13.3%±12.2SDS Vs –5.5%±15.0SDS, *p *< 0.001; *p* < 0.001) and AKI (15.5±11.5SDS Vs –3.9%±15.2SDS Vs, *p *< 0.001).

Comparing the characteristics of patients of both cohorts, we found that patients of the validation cohort were younger and presented lower EHRV, creatinine, highest creatinine/basal creatinine (HC/BC) ratio, sodium, chloride, and hematocrit levels and lower prevalence of AKI, ≥ 5% dehydration, and of the composite outcome defined by ≥ 5% dehydration and/or AKI compared with patients of the derivation cohort (Table 1). When we considered together derivation and validation cohorts, patients with ≥ 5% dehydration had higher EHRV than those without it (21.5% ± 14.1 SDS Vs 4.8% ± 19.0 SDS; *p* < 0.001). Similar findings were found when we compared the EHRV of patients with and without AKI (23.6% ± 13.9SDS Vs 7.3% ± 18.5 SDS; *p* < 0.001).

**Table 1:** Main clinical, demographic, and biochemical characteristics of the derivation and validation cohorts

	**Derivation cohort** **No. = 185**	**Validation cohort** **No. = 151**	***p***
Age, yr, mean (SDS)	9.1 (4.1)	2.9 (2.5)	< 0.001
Male sex, no. (%)	81 (43.8)	81 (53.6)	0.07
HR, beats/min	114.6 (15.5)	113.2 (20.7)	0.49
EHRV	22.9 (13.9)	0.9 (16.6)	< 0.001
Creatinine, mg/dL, median (IQR)	0.79 (0.26)	0.4 (0.1)	< 0.001
HC/BC ratio, median (IQR)	1.37 (0.46)	1.15 (0.54)	< 0.001
AKI, no. (%)	81 (43.8)	37 (24.5)	< 0.001
≥ 5% dehydration, no. (%)	113 (61.1)	52 (34.9)	< 0.001
AKI and/or ≥ 5% dehydration, no. (%)	133 (71.9)	55 (36.4)	< 0.001
Serum sodium level, mEq/L, mean (SDS)	140.0 (3.6)	136.4 (3.8)	< 0.001
Serum chloride levels, mEq/L, mean (SDS)	102.6 (4.6)	99.4 (4.6)	< 0.001
Serum potassium levels, mEq/L, mean (SDS)	4.1 (0.61)	4.4 (0.65)	< 0.001
Haematocrit, %	39.3 (3.4)	35.9 (3.5)	< 0.001

Mean and SDS are shown for normally distributed variables, while median and IQR are shown in case of non-normality

*AKI* acute kidney injury, *EHRV* estimated heart rate variation in acute setting in comparison with 50th percentile of heart rate, *HR* hearth rate, *HC/BC* highest serum creatinine/basal creatinine, *SDS* standard deviation score

**Fig. 4:**
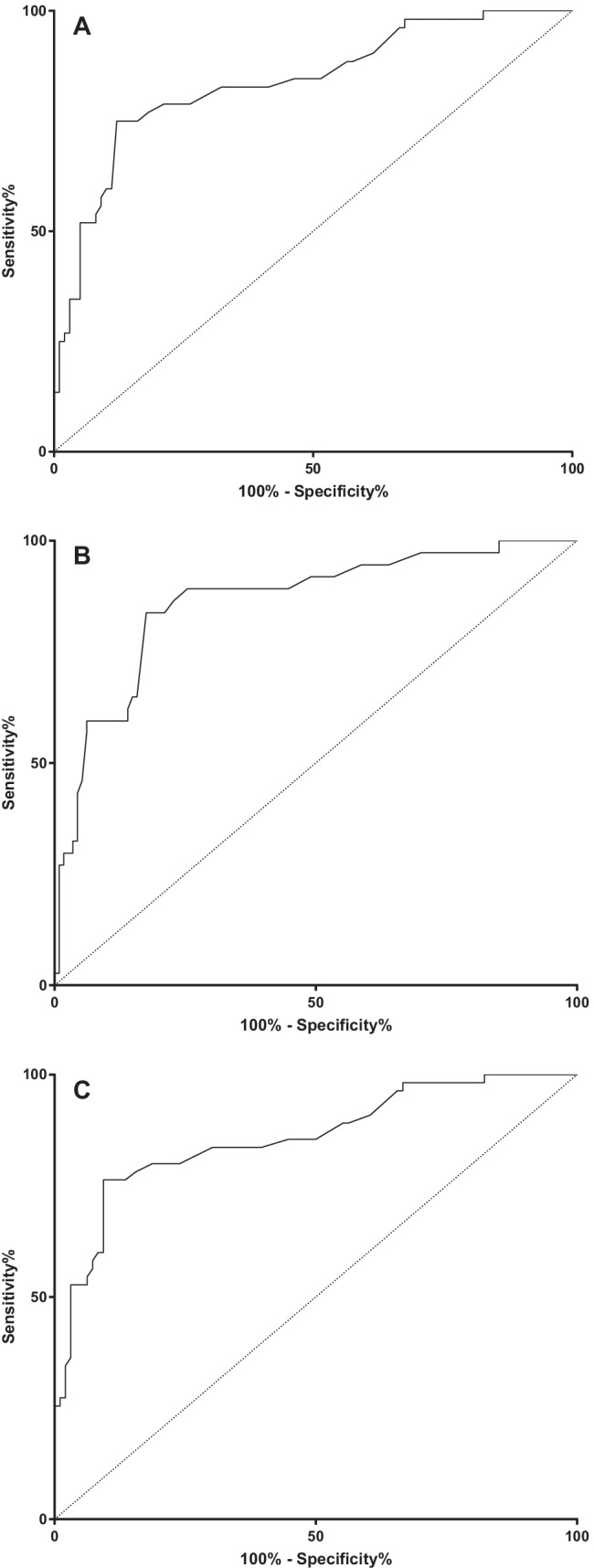
ROC curve analysis of the external validation cohort. **A** ROC curve for ≥ 5% dehydration. **B** ROC curve for AKI. **C** ROC curve for composite defined by outcome ≥ 5% dehydration and/or AKI

We tested in the validation cohort the EHRV cut-off values of > 23.2% for ≥ 5% dehydration, > 24.5% for AKI, and > 24.5% for the composite outcome defined by ≥ 5% dehydration and/or AKI identified in the derivation cohort. The prognostic accuracy of these cut-off values is shown in Table 2. To further confirm the ability of EHRV in discriminating ≥ 5% dehydration from AKI, and children with ≥ 5% dehydration and/or AKI, we also performed ROC curve analysis. The EHRV showed a significant AUROC for ≥ 5% dehydration (AUROC = 0.84; 95%CI: 0.77–0.90; *p* < 0.001) (Fig. 4A), for AKI (AUROC = 0.86; 95%CI: 0.79–0.93; *p* < 0.001) (Fig. 4B), and for the composite outcome (AUROC = 0.86; 95%CI: 0.79–0.91; *p* < 0.001) (Fig. 4C).

**Table 2:** Prognostic accuracy of EHRV cut-offs identified in the derivation cohort for ≥ 5% dehydration and AKI in the validation cohort (gastroenteritis and pneumonia)

**Best cut-offs identified at ROC curve analyses**	**True positive:** **false positive**	**True negative:** **false negative**	**Sensitivity (95%CI)**	**Specificity (95%CI)**	**Accuracy (95%CI)**	**Positive likelihood ratio (95%CI)**	**Negative likelihood ratio (95%CI)**	**Positive predictive value (95%CI)**	**Negative predictive value (95%CI)**	**OR (95%CI)**
**EHRV cut-off > 23.2 as predictor of** ≥ **5% dehydration**	9:1	98:43	21.6% (9.8–38.2)	99.1% (95.2–99.9)	80.1% (72.9–86.2)	24.6 (3.2–190.6)	0.8 (0.7–0.9)	88.9% (50.8–98.1)	79.6 (76.7–82.2)	20.5 (2.5–167.0)*p* = 0.005
**EHRV cut-off > 24.5 as predictor of acute kidney injury**	8:1	113:29	17.3% (8.2–30.3)	99.0% (94.5–99.9)	70.86% (62.9–78.0)	17.1 (2.2–131.6)	0.8 (0.7–0.9)	90.0% (54.0–98.6)	69.5% (66.8–72.1)	31.2 (3.7–259.3)*p* = 0.001
**EHRV cut-off > 24.5 as predictor of acute kidney injury and/or** ≥ **5% dehydration**	9:0	96:46	14.5% (6.5–26.7)	100% (96.2–100.0)	69.5% (61.5–76.8)	Infinity*	0.9 (0.8–1.0)	100%	67.1% (64.7–69.5)	Not calculable*

^*^All the patients with EHRV > 24.5% showed ≥ 5% dehydration and/or acute kidney injury

### Practical application of findings

**Fig. 5:**
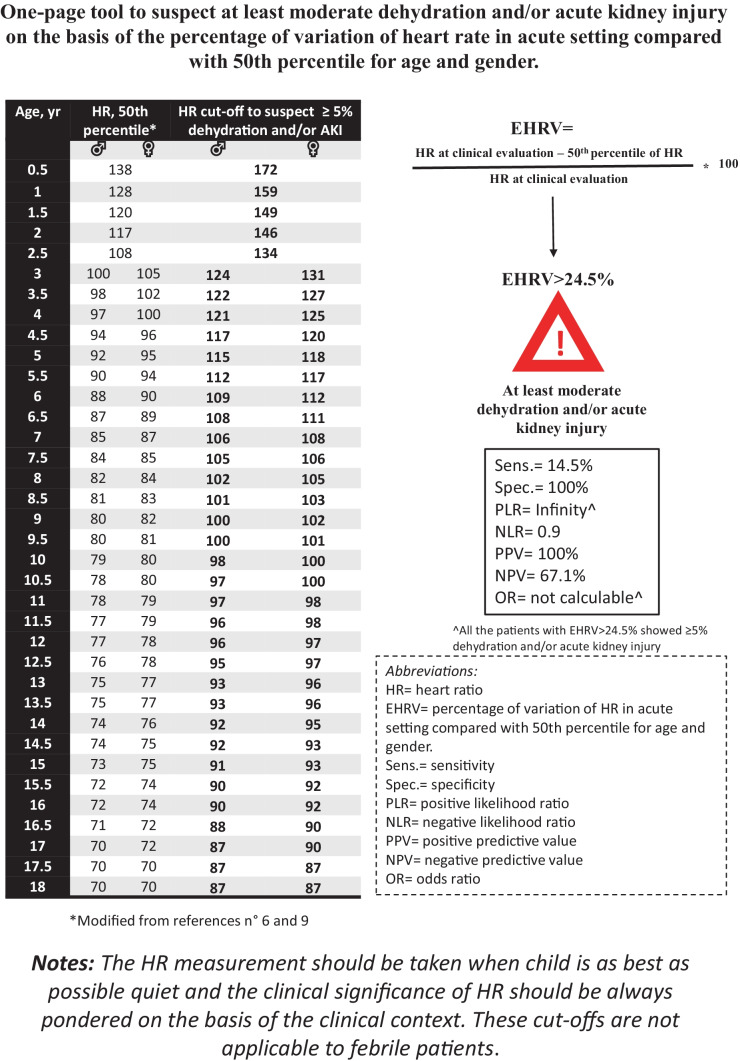
One-page tool to suspect dehydration and/or acute kidney injury on the basis of the percentage of variation of heart rate in acute setting compared with 50th percentile for age and gender

Based on our results, we developed a one-page and EHRV-based tool to help clinicians identify ≥ 5% dehydration and AKI in their daily practice (Fig. 5).

## Discussion

Greater weight loss is associated to higher dehydration degree [4]. As expected, in this study, we found that it is also associated with higher risk of dehydration-related complications. In fact, in our derivation cohort — where we had precise information about weight loss — the greater the degree of weight loss, the higher was the prevalence of AKI.

The lack of correlation between the EWL and MWL confirms the unreliability of parents in defining the weight status of their children [5]. This limits the use of the “anamnestic” weight loss as criterium to define dehydration in a patient admitted to emergency department.

The HR is an easily and quickly detectable parameter. We found that the MHRV is strictly correlated with MWL. In addition, the longitudinally collected data of the DiAKIdney cohort showed that also the EHRV gives an accurate estimation of dehydration degree. This is supported by the significant correlation between MWL and EHRV and by the high accuracy of this latter to identify both dehydration and AKI.

While the diagnostic performance of the EHRV is limited in the DiAKIdney cohort, it significantly improves in the external validation cohort. This difference might reflect that children with T1DM onset are generally more dehydrated. In fact, the mean EHRV, HC/BC ratio, serum sodium and chloride levels, and AKI and ≥ 5% dehydration prevalence were significantly higher in the DiAKIdney cohort compared with validation cohort (Table 1). Another explanation could be that children with T1DM onset present weight loss also for mechanisms different from dehydration such as catabolism related to the lack of insulin. On the contrary, in the general paediatric population — as in children with acute gastroenteritis or community acquired pneumonia — a wide range of dehydration severity is possible and the role of catabolism determining weight loss could be absent. Both the higher dehydration and the possible role of catabolism in the T1DM children could explain the better accuracy of EHRV in the external validation cohort.

The simple record of HR at routine visits (e.g., for growth monitoring) in wellbeing status could be enough to improve our tools to define the dehydration in childhood. Until this becomes part of the current practice, the EHRV can be a useful and easily detectable sign rising the suspect of dehydration and AKI. Our one-page tool (Fig. 5) could help clinicians in their daily practice in order to promptly evaluate the risk of ≥ 5% dehydration and/or AKI. In Fig. 5, we gave particular prominence to the EHRV cut-off which resulted significant predictor of both AKI and composite outcome defined by ≥ 5% dehydration and/or AKI.

AKI, especially if mild in degree, is often overlooked in children, with important future implications given the risk of later chronic kidney disease [11, 14, 23]. EHRV could help the prompt identification of AKI, thus promoting early measures to counteract AKI progression [12]. In fact, extensive preclinical and clinical data show that a timely intravenous rehydration with normal saline can revert the progression of prerenal AKI and avoid subclinical renal damage characterized by renal fibrosis [24, 25].

Our study has some limitations. First, our results are applicable only to dehydrated children without fever. Second, a difference in age between derivation and validation cohort exists. The EHRV, however, performed best in the derivation group indicating a clinical usefulness of EHRV also in younger children. Third, the retrospective nature of the external validation cohort did not allow to accurately discriminate between patients with moderate and severe dehydration from the clinical chart review. Finally, HR is a highly variable parameter and can be affected by fear or anxiety. While in the derivation cohort comprehending a prospectively enrolled cohort of patients, we paid particular attention to the HR detection collecting it when patients were quiet, we cannot assure this attention also in the validation cohort in which the data were retrospectively collected. However, because in the retrospective validation cohort AKI and ≥ 5% dehydration have been defined on the basis of biochemical and of clinical data available from the clinical charts of the patients and because we observed a higher EHRV performance compared with derivation cohort, we can hypothesize that the fear and anxiety of the patients of the validation cohort has not significantly affected our data. In fact, if a patient has fear or anxiety, he/she presents higher HR but without clinical or biochemical anomalies suggesting dehydration and/or AKI so affecting the EHRV performance. On the other hand, the validation using a retrospective cohort could be also a strength of this study allowing us to test our findings using data collected in the daily clinical practice without the “particular attention” to data collection that could be paid in the prospective studies.

In conclusion, our data suggest that the HR could play an important role in the clinical evaluation of a child with suspect of dehydration. The EHRV can predict at least moderate dehydration if > 23.2% and AKI or the composite outcome defined by at least moderate dehydration and/or AKI if > 24.5%.

To avoid misleading translation of our findings in the daily clinical practice, we want to underline that the HR measurement should be taken when child is as best as possible quiet and that the clinical significance of HR should be always pondered on the basis of the clinical context. Further validation of our data in prospectively enrolled cohorts could allow implementing EHRV in the clinical practice.

## Supplementary information

Below is the link to the electronic supplementary material.

Supplementary file1 (DOC 34 KB)

Supplementary file2 (DOC 52 KB)

## Data Availability

The datasets generated during and/or analyzed during the current study are available from the corresponding author on request.

## Abbreviations

AKI: Acute kidney injury
AUROC: Area under ROC curve
EHRV: Estimated percentage of heart rate variation in acute setting compared with 50th percentile of HR according to age and gender
EWL: Percentage of estimated weight loss at T1DM onset
HR: Heart rate
KDIGO: Kidney Disease/Improving Global Outcomes
MHRV: Percentage of measured heart rate variation;
MWL: Measured weight loss at T1DM onset
NPV: Negative predictive value
OR: Odds ratio
PPV: Positive predictive value
ROC: Receiver-operating characteristics
SDS: Standard deviation score
T1DM: Type 1 diabetes mellitus

## Code availability

N/A.
