# Supplementary material for: Heart rate cut-offs to identify non-febrile children with dehydration and acute kidney injury
Source: Eur J Pediatr. 2022 Jan 29;181(5):1967–77. doi: 10.1007/s00431-022-04381-3 (PMC9056451; doi:10.1007/s00431-022-04381-3)
Supplement: Supplementary file 1 — Supplementary file1 (DOC 34 KB) [file 431_2022_4381_MOESM1_ESM.doc]

Article title: Heart rate cut-offs to identify non-febrile children with dehydration and acute kidney injury

**Journal name:** European Journal of Pediatrics

**Authors:** Pierluigi Marzuillo, Anna Di Sessa, Dario Iafusco, Daniela Capalbo, Cesare Polito, Felice Nunziata, Emanuele Miraglia del Giudice, Paolo Montaldo, Stefano Guarino.

**Email address of the corresponding author:** pierluigi.marzuillo@unicampania.it

**Supplementary Text**

***Statistical analysis***

Differences for continuous variables were analyzed with independent-sample t test for normally distributed variables and with Mann-Whitney test in case of non-normality. Qualitative variables were compared by using chi-squared test.

*Derivation cohort*

After log-transformation, a linear regression analysis and a Spearman test was performed to assess the relationship between EWL and MWL, MWL and MHRV, MWL and EHRV, and MHRV and EHRV.

Logistic regression was used to calculate the odds ratio (OR) of presenting with ≥5%, >10% dehydration and AKI (binary variables) both in the unadjusted and adjusted analyses. All variables with p≤0.05 were included in the adjusted analysis. The predictors to be added in these models were defined –on the basis of the available data– before the analyses of this manuscript were performed.

EHRV was evaluated as a potential predictor of ≥5%, >10% dehydration, and AKI by receiver-operating characteristic (ROC) curves analysis. The Youden index was used to identify the best cut-offs [1].

We calculated sensitivity, specificity, accuracy, positive and negative likelihood ratio, positive and negative predictive value (PPV and NPV), and OR of the cut-offs identified at ROC curve analyses.

*External validation cohort*

We tested in this cohort the EHRV cut-off values identified in the DiAKIdney cohort, which were predictive of ≥5%dehydration and AKI. In addition, a new ROC curve analysis was run to evaluate EHRV as predictor of ≥5%dehydration and AKI also in this cohort.

*Development of a practical tool*

We calculated the crude HR value for each age which should increase the clinical suspicion of ≥5% dehydration and/or AKI as follows: 50^th^ percentile of HR for age and sex + [(50^th^ percentile of HR for age and sex/100) * the best EHRV cut-off].

The SPSS software for Windows was used for all statistical analyses except for the ROC curve analysis, which was performed by using Graphpad Prims 7. The calculation of diagnostic performance was calculated by using MedCalc.

**References**

1. Youden WJ (1950) Index for rating diagnostic tests. Cancer 3:32–35.
