# Supplementary material for: Heart rate cut-offs to identify non-febrile children with dehydration and acute kidney injury
Source: Eur J Pediatr. 2022 Jan 29;181(5):1967–77. doi: 10.1007/s00431-022-04381-3 (PMC9056451; doi:10.1007/s00431-022-04381-3)
Supplement: Supplementary file 2 — Supplementary file2 (DOC 52 KB) [file 431_2022_4381_MOESM2_ESM.doc]

Article title: Heart rate cut-offs to identify non-febrile children with dehydration and acute kidney injury

**Journal name:** European Journal of Pediatrics

**Authors:** Pierluigi Marzuillo, Anna Di Sessa, Dario Iafusco, Daniela Capalbo, Cesare Polito, Felice Nunziata, Emanuele Miraglia del Giudice, Paolo Montaldo, Stefano Guarino.

**Email address of the corresponding author:** pierluigi.marzuillo@unicampania.it

**Supplementary Tables**

**Supplementary Table 1.** Univariate and multivariate logistic regression analysis for prognostic factors for ≥5% dehydration, >10% dehydration, or AKI in the derivation cohort (T1DM).

**Supplementary Table 2.** Prognostic accuracy of EHRV cut-offs identified for ≥5% dehydration, >10% dehydration, AKI, and composite outcome AKI and/or ≥5% dehydration in the derivation cohort (T1DM).

**Supplementary Table 1. Univariate and multivariate logistic regression analysis for prognostic factors for** ≥**5% dehydration, >10% dehydration, or AKI in the derivation cohort (T1DM).**

|  | ≥**5% dehydration** | | | | | | | **>10% dehydration** | | | | | | **AKI** | | | | | | |
| --- | --- | --- | --- | --- | --- | --- | --- | --- | --- | --- | --- | --- | --- | --- | --- | --- | --- | --- | --- | --- |
|  | **Univariate** | | | | **Multivariate^c^** | | | **Univariate** | | | **Multivariate^d^** | | | **Univariate** | | | | **Multivariate^e^** | | |
|  | **OR** | **95%CI** | **p** | **OR** | | **95%CI** | **p** | **OR** | **95%CI** | **p** | **OR** | **95%CI** | **p** | | **OR** | **95%CI** | **p** | **OR** | **95%CI** | **p** |
| **EHRV^a^** | 1.4 | 1.1-1.8 | 0.001 | 1.4 | | 1.1-1.8 | 0.005 | 1.6 | 1.2-2.1 | <0.001 | 1.5 | 1.3-2.0 | <0.001 | | 1.5 | 1.2-1.9 | <0.001 | 1.4 | 1.1-1.8 | 0.006 |
| **MWL^b^** | n.a. | n.a. | n.a. | n.a. | | n.a. | n.a. | n.a. | n.a. | n.a. | n.a. | n.a. | n.a. | | 1.1 | 1.06-1.2 | <0.001 | 1.1 | 1.01-15 | 0.02 |
| **Presence of Coma** | 4.9 | 1.4-17.3 | 0.012 | 4.5 | | 1.3-16.2 | 0.02 | 8.0 | 3.0-20.9 | <0.001 | 7.5 | 2.7-20.7 | <0.001 | | 7.6 | 2.5-23.6 | <0.001 | 4.5 | 1.3-15.3 | 0.015 |
| **Ht>45%** | 0.8 | 0.2-3.0 | 0.7 | – | | – | – | 0.3 | 0.04-2.5 | 0.27 | – | – | – | | 1.3 | 0.3-6.8 | 0.7 | – | – | – |

^a^ The odds ratio shown is for every 10% increase in EHR%.

^b^ The odds ratio shown is for ever 1% increase in weight loss.

**Supplementary Table 2. Prognostic accuracy of EHRV cut-offs identified for ≥5% dehydration, >10% dehydration, AKI, and composite outcome AKI and/or ≥5% dehydration in the derivation cohort (T1DM).**

| **Best cut-offs identified at ROC curve analyses** | **True positive:**  **false**  **positive** | **True negative:**  **false**  **negative** | **Sensitivity**  **(95%CI)** | **Specificity**  **(95%CI)** | **Accuracy**  **(95%CI)** | **Positive likelihood ratio**  **(95%CI)** | **Negative likelihood ratio**  **(95%CI)** | **Positive predictive value**  **(95%CI)** | **Negative predictive value**  **(95%CI)** | **OR**  **(95%CI)** |
| --- | --- | --- | --- | --- | --- | --- | --- | --- | --- | --- |
| **EHRV cut-off>23.2 as predictor of**  **≥5% dehydration** | 68:45 | 47:25 | 60.2%  (50.5-69.3) | 66.7%  (54.6-77.3) | 62.2% (54.8-69.2) | 1.8  (1.3-2.6) | 0.6  (0.5-0.8) | 73.9%  (63.7-82.5) | 51.6  (41.0-62.1) | 2.8  (1.5-5.2)  p=0.001 |
| **EHRV cut-off>23.8 as predictor of**  **>10% dehydration** | 38:14 | 80:53 | 73.1%  (59.0-84.4) | 60.9%  (52.1-69.2) | 63.8%  (56.4-70.7) | 1.9  (1.4-2.4) | 0.4  (0.3-0.7) | 42.2%  (31.9-53.1) | 85.3%  (76.5-91.7) | 4.1  (2.0-8.3)  p<0.001 |
| **EHRV cut-off>24.5 as predictor of**  **acute kidney injury** | 50:31 | 68:36 | 61.7%  (50.3-72.3) | 66.3%  (56.4-75.3) | 63.8%  (56.4-70.7) | 1.8  (1.3-2.5) | 0.6  (0.4-0.8) | 58.8%  (47.6-69.4) | 69%  (59.0-77.9) | 3.0  (1.7-5.6)  p<0.001 |
| **EHRV cut-off>24.5 as predictor of ≥5% dehydration and/or**  **acute kidney injury** | 74:59 | 40:12 | 55.6%  (46.8-64.2) | 78.9%  (65.3-88.9) | 61.6%  (54.2-68.7) | 2.6  (1.5-4.5) | 0.6  (0.4-0.7) | 87.1%  (79.6-92.1) | 41.0%  (35.4-46.8) | 4.2  (2.0-8.7) |
